# The Recent Advances in Raman Microscopy and Imaging Techniques for Biosensors

**DOI:** 10.3390/bios9010025

**Published:** 2019-02-12

**Authors:** Alexander Rzhevskii

**Affiliations:** Thermo Fisher Scientific, 2 Radcliff Rd., Tewksbury, MA 01876, USA; alexander.rzhevskii@thermofisher.com

**Keywords:** Raman, spectroscopy, microscopy, imaging, biological cells, SWCNT

## Abstract

Raman microspectroscopy is now well established as one of the most powerful analytical techniques for a diverse range of applications in physical (material) and biological sciences. Consequently, the technique provides exceptional analytical opportunities to the science and technology of biosensing due to its capability to analyze both parts of a biosensor system—biologically sensitive components, and a variety of materials and systems used in physicochemical transducers. Recent technological developments in Raman spectral imaging have brought additional possibilities in two- and three-dimensional (2D and 3D) characterization of the biosensor’s constituents and their changes on a submicrometer scale in a label-free, real-time nondestructive method of detection. In this report, the essential components and features of a modern confocal Raman microscope are reviewed using the instance of Thermo Scientific DXRxi Raman imaging microscope, and examples of the potential applications of Raman microscopy and imaging for constituents of biosensors are presented.

## 1. Introduction

A biosensor is a chemical sensor that uses a biochemical mechanism to convert quantitative or qualitative information about the analyte under study into a detectable signal. Despite the diversity of types and designs [1], the biosensor represents an integral device that consists of two basic components: a highly specific biological recognition element (biochemical receptor) and a transducing structure (detector element) that translates the recognition act into an analytically measurable signal. In recent years, a rapid proliferation of biosensors resulted in multiple reviews dedicated to their constructions, fabrications, performance, and applications [1,2,3,4].

Being the method of the spectral analysis of molecules by essence, Raman microspectroscopy provides extraordinary capabilities for the characterization of biosensors and the processes involved in biosensing mechanisms [5]. Indeed, Raman microscopy has been used for decades and is now well established as one of the most powerful analytical techniques for the analysis of complex biomaterials and monitoring biological processes [6]. From the other side, analytes and the transducing structures of organic and inorganic nature and diverse composition [7], excluding pure metals and metal alloys, can potentially be probed by Raman technique. Progressive miniaturization of biosensors and the usage of nanomaterials [8,9] further highlights the importance of Raman microspectroscopy as a non-invasive method for the spectrochemical characterization of biosensor components. Recently, the synergy between Raman techniques and biodetection systems resulted in a combined cell-based biosensor/Raman microscopy platform [10,11].

Thus, the characteristics, performance, and analytical capabilities of a Raman microscope play an important role in the successful application of the Raman microspectroscopy in biosensing research.

**Figure 1:**
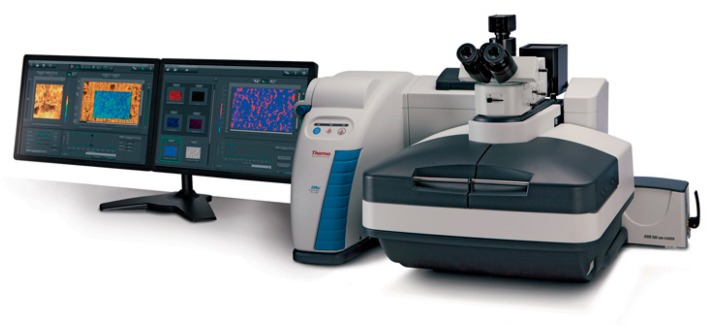
Thermo Scientific DXRxi Raman imaging microscope.

The recent achievements in confocal Raman microscopy and imaging are illustrated by the example of a Thermo Scientific DXRxi Raman imaging microscope (Figure 1) and its usage for the characterization of the materials involved in the biosensing mechanisms or appropriate for bioanalytical devices.

As is typical of Raman microspectroscopy [11], the DXRxi Raman microscope consists of an optical microscope integrated with a Raman spectrometer representing a scientific instrument capable of obtaining both traditional optical and spectrochemical images with a spatial resolution at the diffraction limit of light (less than 1 micron). The high spatial resolution enables the measurement of chemically and spatially complex samples to reveal their chemical composition and inhomogeneity, molecular orientation, conformation, polymorphism, crystallinity, material deformation, local temperature, etc. The optical and spectroscopic imaging can be combined to produce a “hyperspectral” cube, a 3-dimensional set of data providing Raman spectra at every pixel of the 2D area image. By acquiring successive 2D area images at different depths of a given sample in a confocal mode using a motorized XYZ microscope stage, a 3D spectrochemical representation of the sample may be constructed, thus preserving the sample integrity and avoiding possible artifacts that may result from physical cutting or sectioning of the sample.

The developments in the instrument alignment and calibration, excitation options, laser power control, detectors, XYZ sampling stages, and software ensure the DXR Raman microscopes offer a unique combination in the ease of use, performance, flexibility, the speed of analyses, and amount of retrieved spectral information.

## 2. Recent Developments in the Major Components of Modern Raman Imaging Microscope and Selected Examples

### 2.1. Alignment and Calibration

In Raman microscopy, it is important to have the laser beam focused at, and the spectrograph measured from, the same point at the sample. The process of bringing the laser focal point and the spectrograph sampling one into a perfect coincidence is known as *alignment*. If the points appear to be spatially offset at the sample, then the intensity of the Raman scattering radiation received by the spectrograph may be significantly reduced, and spatial discrimination will be affected, thus disabling the Raman microscope to operate in a confocal mode [12].

Over time, all Raman microscopes are subject to alignment drifts. These drifts may be caused by laboratory temperature fluctuations, external disturbances of the instrument, normal wear of system components occurring during usual operation, and many other factors. The misalignments must be corrected by periodic maintenance procedures provided by the Raman microscope’s manufacturer. The regular alignment compensates for variations caused by the inevitable drift over time and guarantees the highest intensity of the collected Raman signal and spatial resolution in the confocal Raman microscope. The Raman microscopes claimed to be “permanently” aligned experience a natural drift in performance over time or in changed conditions of exploitation. 

Calibration is another procedure that ensures the accuracy of experimentally measured Raman spectrum, with the correct Raman shift on the x-axis and Raman intensity on the y-axis. Manually performed alignment and calibration procedures usually require a certain level of qualification of the maintenance personnel, additional materials, and sources, and often increase the downtime of the instrument during the procedures.

The optimal performance and operational readiness of the DXR Raman microscopes are ensured by a fully automated procedure of optical alignment and calibration of all involved optical elements using a patented alignment and calibration tool that excludes errors or subjectivism of a user.

**Figure 2:**
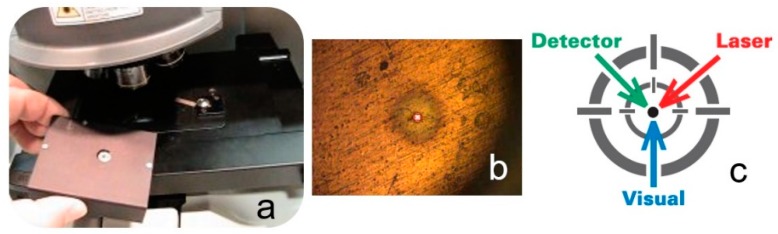
The alignment and calibration tool: (**a**) The tool to be placed on the microscope stage; (**b**) The bright pinhole in the optical focus centered in the binocular cross-hair; (**c**) A schematic diagram showing the optimal alignment of the sampling point, laser excitation, and visual spot.

To align a DXR Raman microscope, the user places the alignment and calibration tool of a matchbox size on the microscope stage and positions it so that the pinhole aperture on the top of tool appears under the microscope crosshairs in the focus of the microscope (Figure 2).

Then, the alignment and calibration procedures are performed in a fully automated software-controllable manner. Inside the box, there is a set of microdevices and materials: a photodiode, a neon lamp, a small piece of polystyrene, and a broadband white light source. During the software-controllable alignment and calibration procedure, a precision micromotor sequentially moves the microparts in and out under the pinhole so that each part, or their combination, participates in essential steps in the alignment and calibration protocol. Namely, the photodiode and the polystyrene are used to align the optical path from the spectrometer and the laser beam, respectively. The correction for the possible wavelength nonlinearity of the spectrograph employs multiple, precisely characterized lines in the emission spectrum from the neon source. The polystyrene standard is used for calibration of the excitation laser frequency, and the multipoint x-axis calibration to adequately correct for distortion and permit spectrograph-to-spectrograph data transfer and reliable library search. The intensity correction uses the standardized broadband light source to compensate for the decline in the silicon-based CCD detector efficiency response at shorter and longer wavelengths.

**Figure 3:**
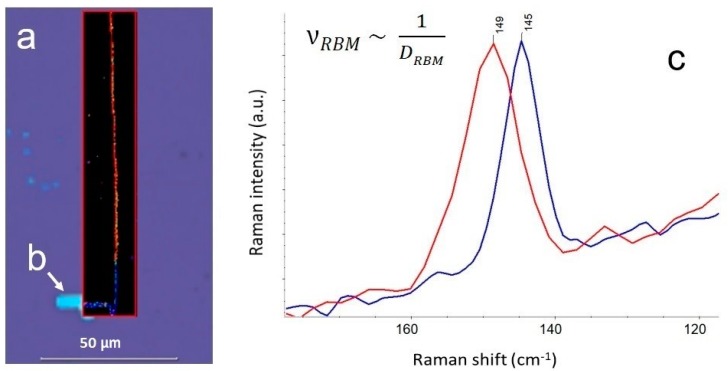
Raman image showing detection of the change in diameter of an individual single-wall carbon nanotube (SWCNT) upon absorbing water: (**a**) the Raman image indicates empty (red) and loaded (blue) parts of SWCNT; (**b**) a micronozzle on a Si substrate used to inject the water; (**c**) the shift of the corresponding radial breathing mode (RBM) peak occurs because the position of the peak is inversely proportional to the diameter of the SWCNT [16,17]. Measurement parameters: 532 nm excitation, 4 mW of the laser power at sample, 100×/0.9 N.A. objective, 50 µm confocal pinhole, 0.4 µm image pixel, 14,000 spectra, 12 min total collect time.

As it is shown in Figure 3, the precise alignment and calibration of DXRxi imaging microscope make it capable of detecting changes in the diameter of an individual single-wall carbon nanotube (SWCNT) by tracking the shift in the position of the peak of so-called radial breathing mode (RBM) upon absorbing water. The unique capability of carbon nanotubes (CNTs) to penetrate biological cell membranes make them a new perspective tool in the field of nanobiotechnology and nanomedicine for delivering therapeutic agents into the cytoplasm and, in many cases, into the nucleus [13,14,15]. While the miserable amount of drug loaded into the individual CNT can hardly be detected directly by any spectroscopic means, the indirect act of exposure of the absorption—by the increase in the CNT’s diameter upon “swallowing” the drug—is observed. The high spatial resolution Raman imaging brings further advantages for tracking and real-time monitoring of drug delivery in vivo.

**Figure 4:**
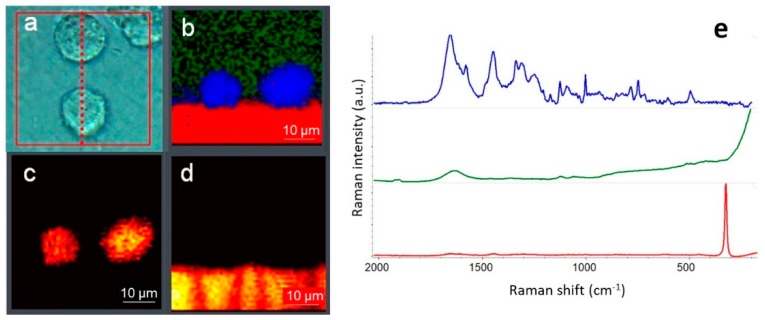
Confocal optical cross-sectioning of biological cells: (**a**) optical image captured with 60× water immersion (WI) objective and the direction of YZ cross-section denoted by the dotted line; (**b**) Raman image generated using multivariate curve resolution (MCR) algorithm (green—buffer, blue—lipids, red—CaF_2_ substrate); (**c**) YZ Raman image generated as integral area of the band at 2925 cm^−1^ assigned to lipids; (**d**) YZ Raman image generated as integral area of the peak of CaF_2_ at 320 cm^−1^; (**e**) average representative Raman spectra of the cell (blue), buffer (green) and CaF_2_ substrate (red) in the “fingerprint” region—the spectra are normalized and offset for clarity. Measurement parameters: 532 nm excitation, 10 mW of the laser power at the sample, 50 µm confocal pinhole, 0.8 µm image pixel, 2500 spectra, 10 min total collect time.

The precise alignment guarantees the highest quality spectral data achievable in the confocal mode of operation (Figure 4).

Regular alignment and calibration ensure optimal performance of the instruments and compensates for variations caused by laboratory temperature fluctuations, instrument disturbances or the other external impacts. 

### 2.2. Laser Options and Power Control

One of the major advantages of Raman spectroscopy is the independence of spectral appearance of an excitation wavelength, which allows an experimenter to consider various excitation wavelengths and find the right one to obtain the most informative Raman data. A suitable laser selection is principally important for the biosensor’s constituents. From one side, Raman microscopy can probe biologically sensitive elements without the need for biomarkers. From another side, biochemical structures like cell and tissues often manifest a relatively weak Raman scattering in the case of conventional non-resonant Raman spectroscopy. Fortunately, the access to multiple excitation wavelengths may allow an ultrasensitive molecular detection through the mechanism of resonance enhancement of Raman signal of the biomarkers or specific molecules found in the biochemical structures.

It is known that fluorescence interference from the constituents of tissue can be greatly reduced in the Raman spectra by using near-IR excitation. Visible excitation Raman spectroscopy is particularly well suited for in situ cellular analysis because of the relatively weaker fluorescence of individual cells compared with intact tissue. In [18], it was emphasized that the excitation wavelengths in the red spectral region are close to maximum transparency in the biological optical window, significantly reducing phototoxicity for conventional as well as for resonance Raman, and enables repeatable spectra acquisition without compromising functions of biological cells and its compartments. Comprehensive reviews of the excitation wavelength strategies for biomedical Raman spectroscopy can be found elsewhere [19,20].

DXR instruments are built on an open configuration principle, allowing the user to select a proper excitation wavelength and to upgrade the instrument immediately in the lab as a new task appears. The option is ensured by the implementation of the user-replaceable lasers, gratings, and rejection filters in the instrument design. There is no limitation imposed on the number of excitation lasers and their characteristics—user-provided lasers can be added to the standard 455, 532, 633, and 785 nm lasers currently offered.

**Figure 5:**
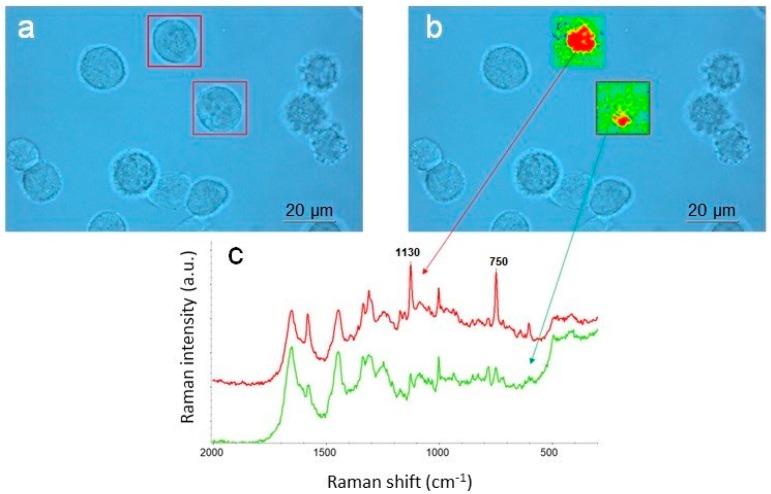
Probing cytochrome *C* in cancerous cells with resonance Raman using 532 nm excitation: (**a**) optical image captured with 60× water immersion (WI) objective; (**b**) Raman images of two cells generated as integral intensities of the resonantly enhanced peak at 1130 and 750 cm^−1^ and superimposed on the optical image; (**c**) representative average Raman spectra of the cytochrome C (red) and the rest of the cell (green)—the spectra are offset for clarity. Measurement parameters: 8 mW of the 532 nm laser power at the sample, 50 µm confocal pinhole, 1 µm image pixel, 925 spectra, 7 min total collect time.

As an example of the excitation wavelengths optimization in a specific experiment, Figure 5 illustrates the detection of cytochrome C in normal and cancerous cells by selective enhancement of the Raman peaks at 1130 and 750 cm^−1^, due to the resonance effect under 532 nm excitation in the prosthetic group that consists of an iron atom contained in the center of a porphyrin [21,22]. The lower right normal cell shows cytochrome C localized in the mitochondria—the red region overlaps the mitochondria in the Raman images of the cell. The upper left cancerous cell, however, shows that cytochrome C is spread throughout the cell, meaning the structure of the mitochondria has been disrupted by cancer.

**Figure 6:**
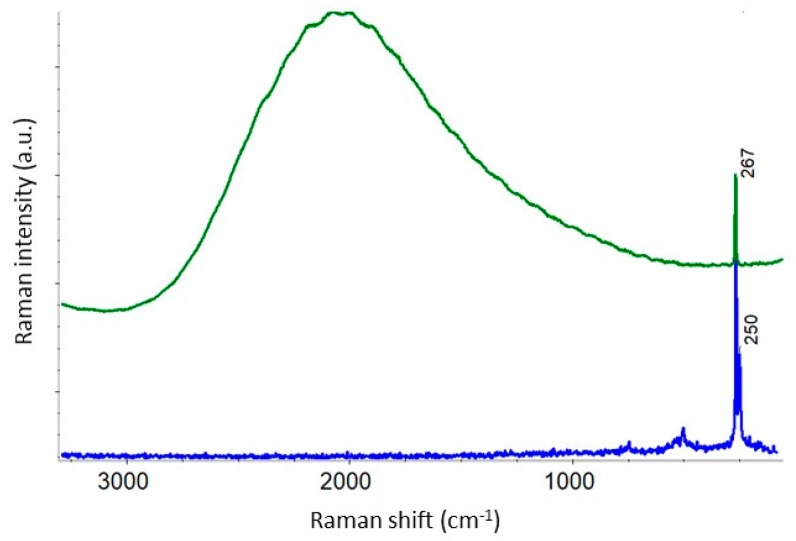
Raman spectra of GaAs obtained with 455 nm (blue) and 532 nm (green) excitations.

Another example of specifically operative excitation is photonic biosensors of various architectures based on GaAs heterostructure [23,24]. The bulk or epitaxial GaAs features a strong intrinsic photoluminescence emission under the excitations from visible green to NIR. Thus, the excitation in a blue region avoiding the emission from the GaAs heterostructure employed for biosensing would mask any contiguous Raman signal that may allow characterization of, for example, the functionalization of the surface of this material by Raman microspectroscopy. Figure 6 compares the Raman spectra of an approximately 1 mm-thick GaAs substrate obtained with 532 and 455 nm excitations.

Since Raman microscopes use a tightly focused laser beam, it is particularly important for light-sensitive constituents of biosensors such as biological, nanocarbon materials, thin films, and small objects to have precise control over excitation laser power to avoid possible damage or alteration of the samples.

DXR instruments are equipped with a finely graduated neutral-density filter delivering accurate and reproducible light power by 0.1 mW increments to the sample. Note that in the example illustrated above in Figure 3, the 532 nm laser power at samples was optimally set to 2.5 mW to avoid any deceptive effects from the laser, yet ensuring high-quality spectral data. 

### 2.3. Spectrograph

The spectrographs widely used for dispersive Raman spectroscopy and based on Czerny–Turner or Echelle arrangements suffer from fundamental limitations. The major issue for a conventional Czerny–Turner spectrograph is astigmatism that results in the spectral resolution varying across the spectrum and with different excitation wavelengths. In modern array detector-based Czerny–Turner spectrographs, a wide spectral range can be obtained by rotating the optical grating. However, the utilization of rotating components usually complicates the calibration procedure and increases calibration time required to maintain accuracy and reproducibility. In addition, the stitched spectrum that the spectrograph generates is subject to potential baseline artifacts at the stitch points and fluctuations in signal-to-noise across each segment of the stitched spectrum. By contrast, Echelle spectrograph has no rotating components and allows the full spectrum to be measured in a single exposure. The drawback of the Echelle spectrograph is that its optical elements are difficult to re-configure for use with multiple excitation wavelengths.

The wideband spectrograph used in the DXR microscopes offers a unique design that enables them to measure the Raman spectrum in the 3500–50 cm^−1^ range at 4 cm^−1^ resolution independent of the excitation laser wavelength in a single exposure using a fixed grating mount [25]. All the fixed diffraction gratings are blazed-angle gratings that concentrate a large percentage of the energy incident on them into a given reflected diffraction order for a specific wavelength. Thus, the efficiency of the gratings optimized for the specified wavelength can reach 80% of absolute efficiency, whereas non-optimized gratings typically do not exceed ~50% efficiency. Since all the lasers in DXR instruments are accompanied by the corresponding blazed gratings, the configuration ensures the high optical throughput and, respectively, the spectral sensitivity at least 1.5 times higher than in non-optimized optical arrangements.

### 2.4. Detector

The rapid occurrence of many processes in biosensors, in combination with the need for low-intensity illumination to reduce the phototoxic impact on biologically sensitive components and living cells, calls for a Raman imaging system that offers high sensitivity and S/N ratios in the spectra at the fastest acquisition times.

DXRxi Raman imaging microscope is equipped with EMCCD (electron multiplied charge-coupled device) detector thermoelectrically cooled down to a low temperature which virtually eliminates dark noise in the detector. EMCCD detectors are different from CCD ones in that they employ a multiplication register to amplify charges. Both EMCCD and CCD have a large wavelength response, typically ranging from 400 to 1000 nm, making them suitable for all lasers except true NIR (1064 nm and longer). Both can be used in the back-illuminated versions, increasing quantum efficiency and, thus, sensitivity. However, the readout speeds of EMCCD detectors are much faster than CCD ones, so they excel at highspeed Raman imaging microscopy with multiple scans. The comparison of the EMCCD and CCD technologies and the types of corresponding detectors can be found elsewhere [26,27].

### 2.5. Sampling Stage

The design and characteristics of the motorized sampling stage in Raman microscopes are important as it determines the way and the rate at which the sample can be scanned, and spectra collected. The collected array of Raman spectra at predefined locations of a sample constitute a Raman map. Conventional instruments use stages driven by stepper motors, while high-speed imaging systems require sampling devices based on raster scanning principle. The main difference is that the former scans the samples in a discrete (point-by-point or stop-and-go) mode while the latter allows scanning continuously without stopping—a critical feature for ultrafast map acquisition.

The raster scanning piezo and galvano scanners are usually limited by a relatively small-sized scanning area. Moreover, the galvano scanners allow for a deviation of the shape of the focused laser beam from an ideal circular spot at the sample plane, thus decreasing the spatial resolution in the off-center regions of the scanned area. Combination of the mechanical stages with the piezo or galvano scanners represents a cumbersome configuration and does not ensure the speed and precision desired for ultrafast map collection. 

DXRxi Raman imaging microscopes employ the proprietary magnetic linear motor stage with closed-loop optical encoders. In contrast to the mechanisms of scanning by a laser beam, the scanning is achieved by moving the stage in a zigzag manner with the laser beam being always orthogonal to the surface of the sample under study, thus keeping the advantage of spatial resolution invariant of the size and position of the scanned area. The stage allows imaging of a sample area up to 3 × 4 in.^2^ with high spatial precision and at a high spectral acquisition rate. 

**Figure 7:**
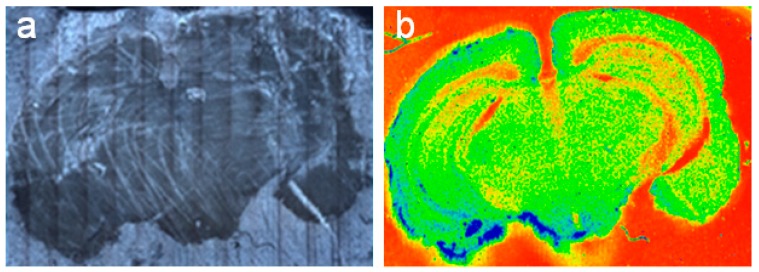
Raman image of a mouse brain tissue section: (**a**) “mosaic” optical image captured with 20× objective; (**b**) color-coded Raman image of 9 × 6 mm^2^ showing variation in the overall intensity of Raman spectra that corresponds to diverse macrostructural as well as microstructural anatomical regions of the brain. Measurement parameters: 24 mW of the 780 nm laser power at the sample, 50 µm confocal pinhole, 25 µm image pixel, 86,994 spectra, 2.5 h total collect time.

Combining the speed of the EMCCD detector with the fast and continuous scanning stage in DXRxi results in hyperspectral imaging of relatively large samples as illustrated in Figure 7.

### 2.6. Software

The main goal of Raman hyperspectral imaging is to provide detailed information about the spatial distribution and morphology of constituents in heterogeneous samples. The Raman image is an image produced by processing the collected Raman map in a variety of ways based on univariate and multivariate statistical models. A scientifically meaningful number of generated Raman images is defined by the distinguishable spectral features assigned to the different sample constituents. Theoretically, the minimal number of Raman images that may be generated from a single map is equal to the number of the resolved spectral elements. 

The “image-centric” and high data volume processing software utilized in DXRxi Raman imaging microscope allows real-time measurement parameter (laser power, exposure time, image pixel size, number of scans, etc.) optimization and Raman image generation on the fly. For example, the real-time application of the multivariate curve resolution (MCR) method in DXRxi, that can be used for immediate imaging of chemical components in heterogeneous samples, cannot be found in any other Raman systems.

**Figure 8:**
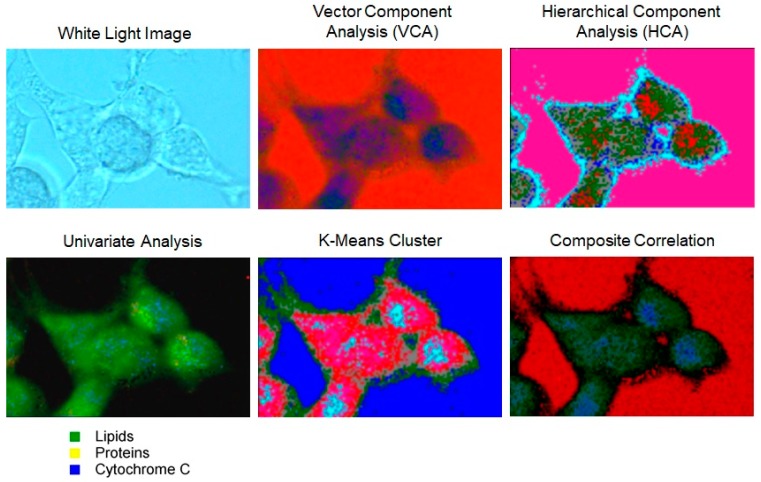
Raman images of live HEK cells were generated using univariate and multivariate methods available in CytoSpec software package and show lipids, proteins, and cytochrome C constituents using different colors. Despite the color schemes varying from one method to another, the images manifest the regions corresponding to the three main constituents. Measurement parameters: 60× WI objective, 10 mW of the 532 nm laser power at the sample, 25 µm confocal pinhole, 0.5 µm image pixel, 1120 spectra, 15 min total collect time.

**Figure 9:**
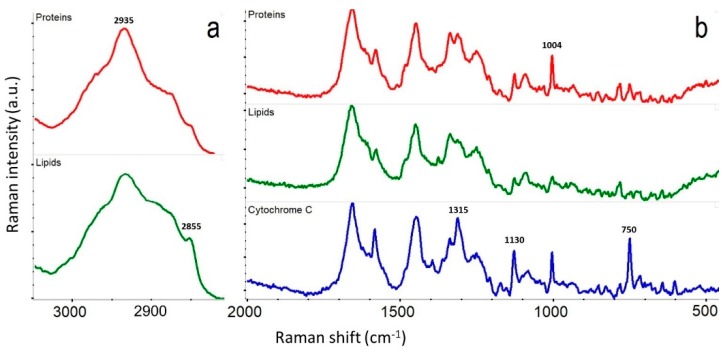
Representative average Raman spectra assigned to the locations of proteins, lipids, and cytochrome C in the live HEK cells shown in Figure 8: (**a**) the spectral region of the CH_x_ stretch vibrations of the hydrocarbon chains with the marked peaks at 2935 and 2855 cm^−1^ associated with proteins and lipids, respectively; (**b**) the “fingerprint” region with the marked peaks at 1004 cm^−1^ assigned to phenylalanine (Phe) typical for proteins, and the resonantly enhanced peaks at 1315, 1130, and 750 cm^−1^ in the cytochrome C.

Raman maps collected in DXRxi can be exported to specialized software packages for hyperspectral image processing such as CytoSpec or ENVI, or transferred to file formats compatible with MATLAB, LabView, Igor Pro, or saved in HDF5 metadata format. The examples of Raman images of biological cells generated using univariate and multivariate processing algorithms, and the Raman spectra retrieved from the locations assigned to the major constituents, such as proteins, lipids, and cytochrome C, are shown in Figure 8 and Figure 9, respectively.

The recently reported BCAbox software, developed and implemented in the DXR Raman microscope [28], further expands the capabilities of Raman microscopy for the precise analysis of molecular content in major cellular organelles.

## 3. Conclusions

Several recent instrumental and technical developments have led to substantial improvements in Raman microscopy and imaging techniques that enhance its potential for systematic applications in biosensor research. Nowadays, commercially available Raman systems utilizing multiple lasers, fast detectors, rapid sample scanning modes, and advanced image processing software can provide effective tools for the characterization of biosensors and its constituents, as well as other molecular systems of any nature at the diffraction limit of light.
